# Epidemiology

**Published:** 1888-04

**Authors:** 


EPIDEMIOLOGY OF THE SECOND QUARTER OF 1887.—The following figures come from the official report of the health-office in Berlin: During the second quarter of 1887, there died in German cities of more than 15,000 people each, 60,294 people, a mortality of 23.6 per 1,000; in those of Austria 45,546, a mortality of 19.8 per 1,000; and in English cities 10,586, a mortality of 19.8 per 1.000. Of persons under one year of age there died in German cities 30.2 per cent. of all deaths, and in English cities 23.3 per cent. In German cities 8,891 died of phthisis, in Austro-Hungarian cities, 6,579. In this quarter typhus carried off 140 in Moscow, 4 in Königsberg, and 1 in Vienna. Typhoid fever carried off in Germany 433 (44 each in Hamburg and Berlin), in Austro-Hungary 287 (14 in Vienna), in Moscow 110, in London 86, in New York 121, in Chicago 64, and in Cairo 184. Diphtheria killed in German cities 2,466 (310 in Berlin, 163 in Hamburg, 114 in Breslau, 63 each in Dresden and Leipzig), 557 in Austro-Hungarian cities (58 in Vienna), 328 in English cities (193 in London), 187 in Moscow, 880 in New York, and 307 in Chicago. Died of scarlatina in German cities 449 (36 each in Berlin and Cologne), Austro-Hungarian cities 360 (97 in Vienna), 596 in English cities (192 in London), 109 in Moscow, 165 in New York. Measles killed in Germany 1,263 (409 in Munich), in Austro-Hungary 667 (298 in Vienna), in

England 3,114 (1,227 in London), in New York 142, and in Chicago 166. Only 29 persons died in large cities of Asiatic cholera (5 in Bombay, and 24 in Madras.) Small-pox killed in Germany 18, in Vienna 23, in Moscow 20, in Lisbon 44, Genoa 36, East India 56 (52 in Bombay), Cairo 16, Alexandria 30, and New York 48. Acute diseases of the respiratory organs killed in German cities 7,555 (750 in Berlin, 325 in Hamburg, 263 in Munich, and 234 in Breslau), in Austria 3,591 (984 in Vienna). Diarrhœa and cholera-morbus killed in German cities 3,551 (1,331 in Berlin), 1,607 in Austro-Hungarian cities, and 576 in English cities.

